# Association between heavy rainfall and flooding events and infectious diseases in Europe: a rapid review, 2018 to 2025

**DOI:** 10.2807/1560-7917.ES.2026.31.31.2500902

**Published:** 2026-08-06

**Authors:** Nina Rise, Aura Georgina Aguirre-Beltran, Anna K Fotakis, Anne Christine Mariager Breschel, Katja Nyholm Andersen, Steen Ethelberg, Giorgia Solda, Agoritsa Baka, Favelle Lamb, Pikka Jokelainen, Jonathan Suk

**Affiliations:** 1 One Health and International Collaborations, Statens Serum Institut (SSI), Copenhagen, Denmark; 2 Department of Emergency and Internal Medicine, Aalborg University Hospital, Thisted, Denmark; 3 Department of Sequencing and Bioinformatics, Statens Serum Institut (SSI), Copenhagen, Denmark; 4 Department of Infectious Disease Epidemiology and Prevention, Statens Serum Institut (SSI), Copenhagen, Denmark; 5 Department of Public Health, Global Health Section, University of Copenhagen, Copenhagen, Denmark; 6 European Centre for Disease Prevention and Control (ECDC), Stockholm, Sweden

**Keywords:** climate change, flooding, heavy rainfall, infection, preparedness

## Abstract

**BACKGROUND:**

Because of climate change, the frequency of extreme weather events, including heavy rainfall and flooding, is increasing in Europe. It is important to understand how such events affect infectious disease incidence to inform preparedness and response activities.

**AIM:**

We aimed to gather evidence of associations between heavy rainfall and flooding and infectious disease incidence in humans in Europe, as well as report preparedness and response measures.

**METHODS:**

In this rapid review, PubMed, Embase and the Cochrane Library were searched using a combination of keywords relevant to infectious diseases as well as to heavy rainfall and flooding to identify published literature and preprints. Articles published between 1 January 2018 and 15 June 2025 were eligible for inclusion.

**RESULTS:**

From 897 records identified, 14 articles were included in the review. In particular, outbreaks of campylobacteriosis and cases of leptospirosis were reported following heavy rainfall and flooding events in Europe. Public health communication on awareness and hygienic practices was among the most commonly reported preparedness and response measure, while structural measures such as sanitary infrastructure and One Health surveillance systems were suggested.

**CONCLUSION:**

While heavy rainfall and flooding have been associated with outbreaks of infectious diseases in different parts of Europe, relatively few studies investigating these associations were identified in Europe despite wide search criteria, highlighting the need for further research to strengthen European preparedness and response measures.

## Introduction

Europe is facing a growing public health challenge. Climate change – driven by greenhouse gas emissions from fossil fuel combustion, deforestation and intensive livestock farming [1] – is not only altering long-term temperature and weather patterns but is also reshaping the continent’s epidemiological landscape. In the past decade, Europe has experienced an increase in extreme weather events, including floods [2]. According to the 2024 European climate risk assessment, Europe is facing escalating extreme weather events, including heavy rain and flooding [3].

These changes can potentially lead to a rising incidence of infectious diseases, including food- and waterborne diseases, such as campylobacteriosis and leptospirosis [4], and may also facilitate the expansion of vector habitats and boost vector replication rates [5]. Additionally, stagnant water following heavy rainfall or flooding offers ideal breeding sites for mosquitoes, raising the risk of vector-borne diseases such as dengue and West Nile fever [6,7].

Heavy rainfall and flooding are among the most frequent natural disasters in Europe, particularly affecting low-lying coastal areas [3]. Warmer air contains more water vapour, which can be released as extreme rainfall [2] or intensify storms [3], especially in central and northern Europe [2,8]. These extreme weather events have been documented across the European continent [3]. Fluvial floods can transport animal faeces from soil into water supplies and expose people to zoonotic pathogens, while pluvial floods in urban areas may cause sewage overflows that can expose populations to waterborne pathogens and gastrointestinal infections. Moreover, infrastructure damage from such events can indirectly facilitate the spread of infectious diseases [5,6].

A previous European-wide review, reporting on articles published from 2005 to 2018, linked flooding with leptospirosis, anthrax, as well as infections with West Nile virus, *Cryptosporidium hominis*, *Escherichia coli*, *Campylobacter jejuni*, *Giardia lamblia*, Sindbis virus, Batai virus, Tahyna virus and norovirus, and also with unspecified gastrointestinal, influenza-like illness and dermatological complaints [5]. The European continent has experienced several heavy rainfall and flooding events since 2018, calling for an update of the evidence base to inform and guide infectious disease preparedness and public health response. This article seeks to do so by presenting findings from a rapid review examining the associations between heavy rainfall and flooding events and infectious diseases among humans in Europe.

## Methods

We conducted a rapid review following the Cochrane Rapid Review Interim Guidance [9]. The scope of the rapid review was as follows:

- Heavy rainfall and flooding. This rapid review focused on heavy rainfall and flooding. These events are among the most common extreme weather events in Europe and are known to be associated with infectious diseases [5]. To avoid excluding potentially relevant studies, we did not use a specific definition of what constituted a heavy rainfall or flooding. Instead, various definitions for heavy rainfall and flooding were accepted, aligned with existing definitions [10-13].
- Infectious diseases. This rapid review included articles reporting on any type of infectious disease. Articles reporting on diseases caused by specific pathogens, as well as infectious diseases of unknown aetiology identified on the basis of symptomatology e.g. gastrointestinal infection, were eligible for inclusion, as were articles reporting on outbreaks and disease patterns identified through long-term surveillance data.
- Europe. This rapid review included all countries in the European Union (EU), the European Economic Area (EEA), the United Kingdom (UK) and EU enlargement countries. The concept of geographic scope was not part of the database search string.

### Search strategy

PubMed, Embase and the Cochrane Library were searched to identify published literature as well as preprints. Search strings applied a combination of keywords in English relevant to infectious diseases as well as to heavy rainfall and flooding (for detailed search strings, please refer to Supplementary Table S1).

### Inclusion criteria and screening

Studies on infectious diseases and heavy rainfall or flooding published between 1 January 2018 and 15 June 2025 were eligible for inclusion. Inclusion criteria were studies reporting on the association or impact of heavy rainfall or flooding on infectious diseases among humans in Europe. Reference lists of included articles and identified reviews were searched for further relevant references. Additional articles suggested by public health experts in the field of waterborne diseases from ECDC and SSI were considered. Only articles reporting on original data were included in the analysis.

The online platform CADIMA (Julius Kühn-Institut, Quedlinburg, Germany) was used for screening records [14]. Consistency checks were performed to ensure an inter-rater agreement of at least 90%.

### Data extraction and quality control

Data extraction was performed using a piloted data extraction sheet. The quality of the articles was assessed using a modified version of the Joanna Briggs Institute Critical Appraisal Checklist for Analytical Cross Sectional Studies [15], where questions six and eight were omitted to suit the rapid review approach (for the modified tool, see Supplementary Table S2). References were managed using EndNote bibliographic software (Clarivate Analytics, Philadelphia, United States (US)).

## Results

**Figure 1:**
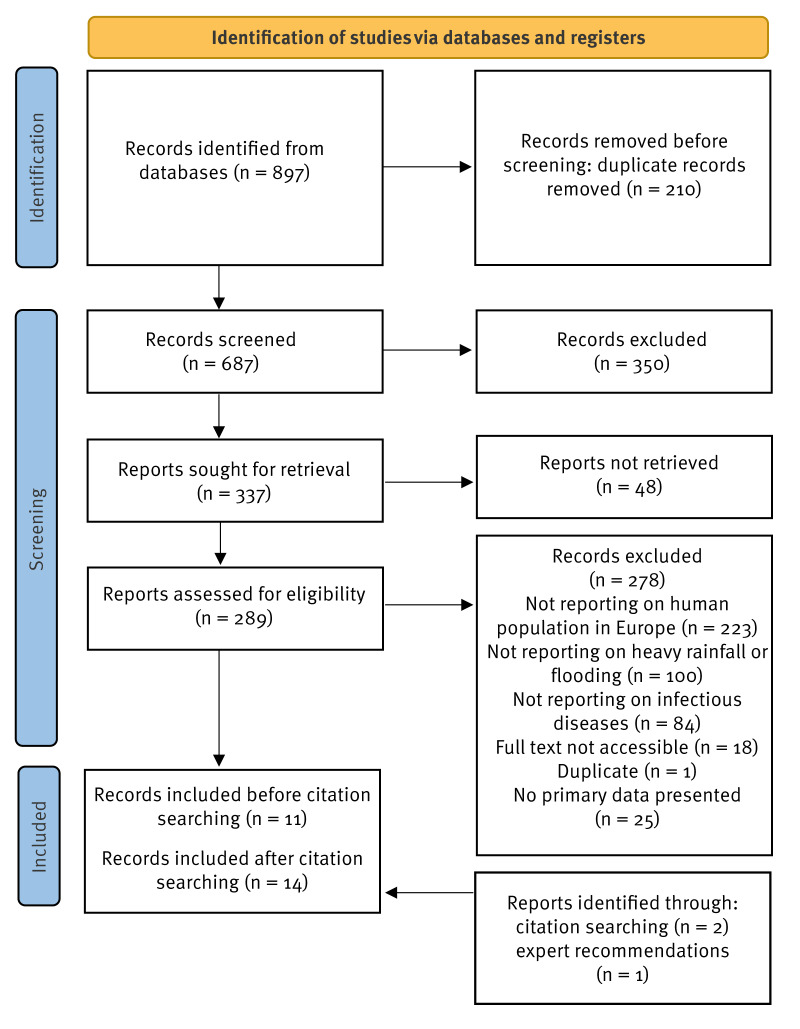
Flow diagram of article selection for the rapid review on infectious diseases and heavy rainfall or flooding events, European Union/ European Economic Area countries, the United Kingdom and European Union enlargement countries, 1 January 2018–15 June 2025

Adapted from PRISMA 2020 flow diagram, source: Page MJ, et al. BMJ 2021;372:n71. https://doi.org/10.1136/bmj.n71.

In total, the database searches yielded 897 records, of which 289 full texts were assessed after title and abstract screening. After full text screening, a total of 11 articles were included. Three additional articles were identified: two through citation searching and one through expert recommendations, bringing the total number of included articles to 14 (Figure 1).

**Figure 2:**
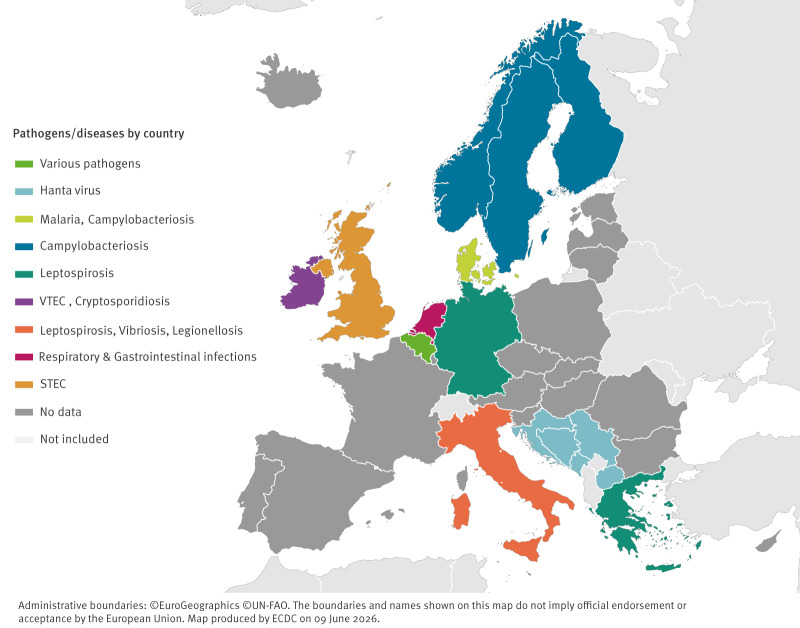
Map to show geographical distribution of reported diseases or pathogens associated to heavy rainfall or flooding events, European Union/ European Economic Area countries, the United Kingdom and European Union enlargement countries, 1 January 2018–15 June 2025

STEC: Shiga toxin-producing *Escherichia coli*; VTEC: verocytotoxigenic *Escherichia coli*.

In total, eight articles were outbreak or case reports [16-23], five provided a longer-term perspective (four of these by analysing surveillance data [24-27] and one by using 19th-century data on malaria in Denmark [28]) and one was a modelling study, forecasting the impact of climate change on campylobacteriosis incidence in the Nordic countries [29]. The included articles reported on campylobacteriosis [17,24,29], leptospirosis [18-20], Shiga toxin-producing *E. coli* (STEC) infection and verocytotoxigenic *E. coli* (VTEC) infection [16], vibriosis [21], hantavirus infection [25], legionellosis [22], cryptosporidiosis [26], malaria [28], various infectious diseases [27] and symptoms of acute gastrointestinal and respiratory infections [23]. The geographic distribution of reported diseases or pathogens associated with heavy rainfall or flooding events is illustrated in Figure 2.

### Disease outbreaks and case series

In total, eight articles [16-23] reported outbreaks or case series of infectious diseases following heavy rainfall or flooding events. Reported clinical and demographic information is summarised in Table 1. An outbreak investigation into 259 cases of STEC in the United Kingdom (UK) combined whole genome sequencing, epidemiological analysis and food chain investigations with meteorological analysis [16]. Based on the epidemiological analysis and food chain investigations, lettuce from one specific grower was found to be the likely source. Meteorological analysis showed that the area where the grower was located had experienced heavy rainfall events 2 weeks before the peak of symptom onset among cases. The rainfall followed a period of drought in the area. Furthermore, geospatial analysis showed sheep holdings in the area. While no link could be established as the livestock was not tested for *E. coli*, the authors speculated that pathogens from the sheep holdings may have been flushed into the lettuce field by the rainfall [16]. One study investigated an outbreak of 1,573 cases of campylobacteriosis that occurred in Norway in 2019 following heavy rainfall [17]. Contamination of drinking water occurred through cracks in a mountain reservoir, which were probably caused by the heavy rainfall event following an extended dry period. The risk of infection was 4.6 times higher among residents receiving water from a specific water reservoir. While unable to draw definite conclusions, the authors suspected that water in the reservoir was most likely contaminated through these cracks during the heavy rainfall.

**Table 1:** Reported clinical and demographic information of cases described in outbreak reports and case reports included in this rapid review, European Union/ European Economic Area countries, the United Kingdom and European Union enlargement countries, 1 January 2018–15 June 2025

Article (first author et al., country, year)	Disease / syndromes	Reported number of cases	Case description	Reported deaths (case-fatality rate)	Identified determinants of vulnerability	Reported risk factors
Cunningham et al., UK, 2024 [16]	Shiga toxin-producing *Escherichia coli* (STEC) infection	259	55% of cases were female. More than 50% of cases were between 20 and 39 years old. Clinical information was available from 255 cases. Reported symptoms were: diarrhoea (92%); abdominal pain (87%); bloody stools (65%); nausea (53%); vomiting (32%); fever (30%). No cases developed haemolytic uraemic syndrome. 30% of cases were hospitalised.	0 (0%)	NA	Eating out, eating salad, eating chicken, eating beef.
Hyllestad et al., Norway, 2019 [17]	*Campylobacter* infection	1,573	Reported symptoms were: diarrhoea (89%); abdominalia (74%); headache (52%); nausea (51%); fever (48%); abdominal distention (35%); vomiting (16%); bloody stools (6%).	0 (0%)	NA	Drinking contaminated water.
Poulakida et al., Greece, 2024 [18]	Leptospirosis	13	54% of cases were female. Mean age: 40.2 years old. Reported symptoms were: rash (69%); fever (62%); myalgia (31%); renal failure (15%); jaundice (8%); haematoma (8%). 11 of 13 cases reported contact with floodwater, of these 9 occurred in domestic settings while two were occupationally exposed (an electrician and a construction worker, both carrying out related repair work). 15% of cases were hospitalised.	0 (0%)	Younger people, due to higher risk of exposure. Older people, due to higher risk of severe disease.	Direct contact with floodwater.
Dreesman et al., Germany, 2022 [19]	Leptospirosis	45 (13 confirmed, 32 probable)	All cases were strawberry harvesters, many originating from neighbouring countries. 60% of cases were male. The age range was 18–50 years old, with a mean of 25 years. Reported symptoms were: fever (89%); myalgia (64%); headache (64%). 47% of cases were hospitalised.	0 (0%)	Field workers, due to higher risk of exposure. Migrant workers, due to language barriers.	Field work.
Vitale et al., Italy, 2018 [20]	Leptospirosis	2	Both cases were female, aged 67 and 71 years old, respectively. Both reported a lower-middle class income. Reported symptoms were: Case 1: jaundice, hepato-splenomegaly, renal failure; Case 2: fever, common malaise; rash, jaundice, hepato-splenomegaly, with disease progression to renal failure and meningitis. Both cases were hospitalised.	1 (50%)	Persons with low-income.	NA
Zaghi et al., Italy, 2024 [21]	Vibriosis	5	Three males and two females aged 74 to 90 years old, presented with skin or soft tissue infection, ranging from cellulitis to necrotising fasciitis. Four cases had reported current comorbidities, and the fifth a history of colorectal cancer. All cases were hospitalised and treated with relevant antibiotics, and in two cases also surgery. One case was admitted to the intensive care unit. Two fatalities were reported in two cases with known skin lesions (Case 1: female, 85 years old, known chronic heart failure and chronic ulcers on the lower limbs; Case 2: male, 77 years old, known atopic dermatitis treated with steroids).	2 (40%)	Older age, especially if having comorbidities, or skin lesions.	NA
Faccini et al., Italy, 2019 [22]	Legionellosis	52	38% of cases were female. Cases ranged in age from 33 to 95 years old, with a mean of 75 years. 41 cases were reported to have an underlying disease, with 22 having two or more chronic conditions. Eighteen were smokers. 77% of cases were hospitalised.	5 (10%)	NA	NA
Mulder et al., the Netherlands, 2019 [23]	Acute gastrointestinal infections and respiratory tract infections	N/A	Of 1,158 questionnaire respondents, 40% were female (sex unknown for 23%) with a median age of 47 years. 608 respondents reported they had been exposed to floodwater and 505 reported they had not been exposed. Of the exposed, 12% reported symptoms of acute gastrointestinal infection and 21% reported symptoms of acute respiratory infection. Of the not exposed, 2% reported symptoms of acute gastrointestinal infection and 7% reported symptoms of acute respiratory infection.	0 (0%)	NA	NA

NA: not applicable.

There were three studies on cases of leptospirosis associated with heavy rainfall and flooding [18-20]. In the first study, 13 cases of leptospirosis occurred following severe flooding in Thessaly, Greece [18]. All cases had confirmed exposure to flooded environments. Two of the cases were classified as being in high-risk occupations (not further specified). Contact with floodwater was confirmed for 11 cases e.g. through clean-up activities or inspecting the surroundings of their homes [18]. The authors noted that seven of the cases were female, in contrast to leptospirosis historically more commonly affecting males. In the second study, 45 cases of leptospirosis occurred among workers on two strawberry farms in Germany during the summer of 2014 [19]. The outbreak was preceded by a period of heavy rainfall (50 mm) and above-average temperatures (17–25 °C). In the third study, a case series of two cases of leptospirosis with suspected link to heavy rainfall were reported on the island of Sicily, Italy in 2009. One case had been in contact with urban street floodwater while driving their car following heavy rainfall [20], and the second case had cultivated vegetables in a garden on the bank of a river where heavy rainfall had caused water levels to rise and riverbanks to flood, although no direct exposure to floodwater could be established for that case.

A case series described five cases of vibriosis caused by *Vibrio vulnificus* (n = 3) and *Vibrio harveyi* (n = 2) occurring within 1–3 months following severe flooding in Italy’s Romagna region where mud and river water had poured into the sea [21]. All cases were elderly people who had chronic conditions predisposing them to infection. Four of the cases had confirmed exposure to seawater in the Romagna region, while the exposure could not be confirmed for the fifth case whose symptoms occurred 3 months after the floods. A case-crossover study and a case-control study were conducted in relation to an outbreak of 52 cases of legionellosis in Bresso, Italy in the summer of 2018 [22]. The case-crossover study found that extreme precipitation 5–6 days before symptom onset was associated with a fourfold increased legionellosis risk while the case-control study found a significant eightfold increase in risk for people living near the same public fountain. All cases were laboratory-confirmed by a urinary antigen test for *Legionella pneumophila*. Outbreak investigators found a public fountain to be the most likely source of infection. The summer was unusually warm (35 °C on average) and humid with two heavy rainfall events. While unable to draw definite conclusions, the authors speculated that the combination of high temperatures, high humidity and heavy rainfall events created optimal conditions for *Legionella* growth in the fountain.

A retrospective cross-sectional survey in 60 locations impacted by floods was conducted 2 weeks after urban pluvial flooding events in the Netherlands in the summer of 2015 [23]. Questionnaires collecting data on self-reported symptoms of gastrointestinal infection and respiratory tract infection and information on floodwater exposure in the previous 2 weeks were sent to households in these locations. In total 3,382 households were invited to participate and 699 (21%; 1,656 individual participants) completed the questionnaire. The study found significant associations between contact with floodwater and symptoms of gastrointestinal and respiratory tract infection symptoms. Information on causative pathogens was not collected, but risk factors for disease were found to be post-flood cleaning both indoors and outdoors, non-cleaning contact with floodwater, cycling through floodwater and having skin contact with floodwater.

### Long-term studies

Five studies investigated the association between heavy rainfall and flooding in a long-term perspective [24-28].

A retrospective analysis of 60,725 campylobacteriosis cases in Denmark from 2000 to 2015 described four community-wide outbreaks involving at least 1,300 individuals [24]. Three of the four outbreaks were related to heavy rainfall or flooding. Two outbreaks were caused by contaminated drinking water following heavy rainfall that caused backflow of sewage into drinking water reservoirs. The third outbreak was related to athletes competing in a triathlon, where heavy rainfall caused sewer overflow polluting the seawater in which the athletes swam.

A study investigating the epidemiology of 1,065 cases of haemorrhagic fever with renal syndrome caused by hantavirus across Bosnia and Herzegovina, Croatia, Montenegro, North Macedonia and Serbia from 2006 to 2015 found a seasonal variation in incidence with a yearly peak during the summer months [25]. In 2014, incidence further increased in May following a cyclone that caused heavy rainfall and severe flooding events in all five countries. There was a strong negative correlation between monthly incidence and the number of months after the cyclone.

A retrospective, ecological study from Ireland compared spatial VTEC (same organism as STEC) and *Cryptosporidium* spp. incidence 2013–2018 with incidence 2015–2016; a period covering repeated flooding events during November and December 2015 [26]. Yearly peaks in incidence usually occurred in the late summer months, however, in April 2016, a peak of 23 residual cases in addition to the seasonal trend was observed. Secondary residual peaks were also observed during June and July 2016 [26]. Both during and after the flood period, risk of VTEC and *Cryptosporidium* spp. was higher in flood-prone areas [26].

A study from Belgium analysing epidemiological data from 2011 to 2021 found associations between heavy rainfall events and incidence of various infectious diseases [27]. However, the association was only statistically significant for infection with respiratory syncytial virus (RSV), where incidence increased after heavy rainfall following a 1-week lag.

A historical example linking a flooding event to an outbreak was documented in Denmark in the late 19th century [28]. In November 1872, a major storm surge inundated low-lying areas and islands in the south-east of the country, with Falster the most heavily affected. In the subsequent year, 1873, a marked increase in *Plasmodium vivax* malaria was reported, with incidence on Falster tripling in spring compared with the previous year [28]. A smaller increase was noted in neighbouring Lolland, while no increase in incidence occurred elsewhere in Denmark; the observed pattern suggests that the flooding event may have played a role in amplifying malaria transmission.

### Modelling studies

A modelling study investigated the link between *Campylobacter* infection and heavy rainfall in four Nordic countries, Denmark, Finland, Norway and Sweden using register data from the period 2000–2015 during which the countries reported a combined total of 64,034 cases [29]. It found that during summer periods, *Campylobacter* cases increased significantly following a 1-week lag after heavy rainfall, while heavy rainfall was associated with a decrease in *Campylobacter* cases during winter months. When analysing the data at municipal level, the study found that following a 1-week lag, for each 1 mm increase in precipitation during the summer months, *Campylobacter* cases would increase by 38%, and for each additional heavy rainfall event, the increase would be 50%. Using precipitation, temperature and demographic projections, the authors estimated that from the baseline period of 2000–2015, the average *Campylobacter* infection incidence would increase from 42 per 100,000 population to 51 per 100,000 population in 2040–2049 and potentially to 117 per 100,000 population in 2080–2089. The authors note that long-term predictions are associated with a high degree of uncertainty in the calculations.

### Cascading pathways

Three of the included articles provided examples of cascading pathways through which heavy rainfall led to outbreaks of infectious diseases [16,17,24]. Combining human, animal, land-use and meteorological data in a STEC outbreak investigation in the UK, it was hypothesised that heavy rainfall washed pathogens from an animal pasture into fields with crops, causing direct contamination through surface flooding [16]. Similarly, incorporating environmental and meteorological data in an investigation into a large outbreak of campylobacteriosis in Norway, heavy rainfall following an extended dry period was suspected to have caused cracks in the walls of a water reservoir, leading to contamination of drinking water [17]. A retrospective analysis of 15 years of epidemiological data on campylobacteriosis in Denmark linked three outbreaks to heavy rainfall events [24]: in two outbreaks, heavy rainfall had caused backflow of sewage into drinking water reservoirs and in the third outbreak, heavy rainfall caused sewage overflow, leading to severe pollution of the sea and coinciding with a triathlon competition where athletes swam in the sea.

### Implemented and recommended preparedness and response measures

Preparedness and response measures used or suggested in the articles describing outbreak or case reports are summarised in Table 2.

**Table 2:** Applied or suggested infectious disease preparedness and response measures in relation to heavy rainfall and flooding, included in this rapid review, European Union/ European Economic Area countries, the United Kingdom and European Union enlargement countries, 1 January 2018–15 June 2025

Article (first author et al., country, year)	Disease / syndromes	Applied preparedness and response measures					Suggested preparedness and response measures (lessons learnt)					
		Raise healthcare professionals’ awareness of the post-flooding /-heavy rainfall risk	Targeted advice for occupationally exposed persons on symptoms, seeking care and use of personal protective equipment	Community-wide health education and information on hygiene measures e.g. hand washing or safe cooking	Health education e.g. information on symptoms and guidance on seeking care	Other	Raise healthcare professionals’ awareness of post flooding/heavy rainfall risk	Targeted advice for vulnerable groups or groups with high risk of exposure e.g. on use of personal protective equipment	Develop One Health early warning systems comprising human, animal, environmental and meteorological data	Community-wide health education e.g. on hand hygiene	Improved sanitation / sanitary infrastructure	Use syndromic surveillance to supplement and support diagnostic testing in case finding
Cunningham et al., UK, 2024 [16]	Shiga toxin-producing *Escherichia coli* (STEC) infection	Yes	NA	Yes	Yes	Yes^a^	NA	NA	Yes	NA	NA	NA
Hyllestad et al., Norway, 2019 [17]	*Campylobacter* infection	NA	NA	Yes	NA	NA	NA	Yes	NA	NA	NA	NA
Poulakida et al., Greece, 2024 [18]	Leptospirosis	NA	NA	NA	NA	NA	NA	NA	NA	Yes	NA	NA
Dreesman et al., Germany, 2022 [19]	Leptospirosis	Yes	Yes	NA	NA	NA	NA	NA	NA	NA	NA	NA
Vitale et al., Italy, 2018 [20]	Leptospirosis	NA	NA	NA	NA	NA	NA	NA	NA	Yes	Yes	NA
Zaghi et al., Italy, 2024 [21]	Vibriosis	NA	NA	NA	NA	NA	Yes	Yes	NA	NA	NA	NA
Faccini et al., Italy, 2019 [22]	Legionellosis	NA	NA	NA	NA	Yes^b^	NA	NA	NA	NA	NA	NA
Mulder et al., the Netherlands, 2019 [23]	Acute gastrointestinal infections and respiratory tract infections	NA	NA	NA	NA	NA	NA	NA	NA	NA	Yes	NA

NA: not applicable; UK: United Kingdom.

^a^ Review of the Hazard Analysis and Critical Control Points (HACCP) plan of a lettuce grower suspected to be the source of the outbreak.

^b^ Deactivation, cleaning and disinfection of public fountain suspected to be the source of the outbreak.

## Discussion

This study, compiling evidence from a rapid review, shows that heavy rainfall and flooding have been associated with the occurrence of various infectious diseases in different parts of Europe in the past decade. In the included articles, campylobacteriosis [17,24,29] and leptospirosis [18-20] were the diseases most commonly reported on in the context of heavy rainfall and flooding. Moreover, four investigations analysing multi-year epidemiological data consistently demonstrated a pattern linking flooding events to subsequent infectious disease outbreaks [24,25,27,29]. Public health communication was reported in three articles as a preparedness and response measure applied in relation to heavy rainfall and flooding events.

This work revealed that published research on the topic since 2018 is relatively limited. Thus there is a need to emphasise the importance of event reporting and research to increase the evidence base in this area, especially as the frequency of extreme weather events has increased and is expected to continue to do so in Europe and globally. Until a more comprehensive body of evidence is established, it remains uncertain how well the current evidence captures the magnitude and complexity of the association between these extreme weather events and infectious diseases. This limitation calls for a concerted effort to prioritise interdisciplinary research between environmental and public health sectors including integrated One Health approaches to address this critical intersection of climate change and infectious disease dynamics [2]. There is a need to better understand these associations, as environmental changes have an impact on public health outcomes as well as on the measures needed to mitigate these risks.

The evidence base identified in this rapid review is skewed towards publication from north-western Europe, which may reflect publication and language patterns, with nine of 14 identified publications reporting from Belgium [27], Denmark [24,28,29], Finland [29], Germany [19], Ireland [26], the Netherlands [23], Norway [17,29], Sweden [29] and the UK [16]. Of the other five articles, three reported on Italy [20-22], one on Greece [18] and one, a multi-country study, reported data from Bosnia and Herzegovina, Croatia, Montenegro, North Macedonia and Serbia [25]. To fully assess the impact of heavy rainfall and flooding events on infectious diseases in Europe and be able to develop geographically effective and equitable preparedness and response measures, more knowledge of how southern, central and eastern Europe is impacted is warranted. The articles found in this rapid review more often concerned specific events, such as outbreaks, rather than broader analysis of this thematic area. In this sense, public health professionals would benefit from syndromic surveillance tools and other specific intersectoral guidance to address the needs of responses to flooding events.

In a review from 2020, one of the most commonly reported preparedness and response measures following flooding in Europe was public health communication regarding awareness and hygiene practices targeted at the public, people occupationally exposed to floodwater, and healthcare professionals. In addition, structural measures such as One Health surveillance systems and improved sanitary infrastructure were reported but to a lesser extent [5]. Given the diversity of disease outbreaks reported and of cascading pathways identified by this review, there is a need for coordinated preparedness and response planning in Europe linking health, environment, and civil protection authorities, to ensure that provisions for monitoring and mitigating infectious disease risk are integrated into flooding response plans. Such plans should include provisions for implementing scalable and setting-specific approaches - such as syndromic surveillance - as traditional disease surveillance systems are not typically designed to detect outbreaks linked to environmental events. Relatedly, developing integrated One Health systems incorporating human, animal, environmental and meteorological data could allow for early warnings linked to environmental signals, as well as earlier detection and response in case of heavy rainfall and flooding events [16].

The preparedness and response measures reported to have been applied in relation to heavy rainfall or flooding events were primarily in the form of public health communication, targeting healthcare professionals [16,19], occupationally exposed persons [19] and the general public [16,17]. However, the importance of developing communication specifically targeting vulnerable groups in the face of future heavy rainfall and flooding events was highlighted [17,21]. This could include groups vulnerable because of socioeconomic factors [20,30] or because of physiological factors such as older age [18,21,30], having comorbidities [18,21] or living with a disability [30] as well as groups with a high risk of being exposed to floodwater, such as young people [18].

While health education and awareness raising are key public health measures, they should be supplemented with structural measures to mitigate the hazard posed by heavy rainfall and flooding, such as urban sanitation, especially in urban slums, to reduce the risk of contamination [20] and drainage systems to prevent pluvial flooding [23].

The rapid review approach proved suitable for the topic of climate change and infectious diseases and the search strategies can be reapplied to identify new studies. However, limitations of the rapid review approach need to be emphasised: (i) some relevant studies may have been missed, (ii) heavy rainfall and flooding were defined differently across the included studies posing a challenge when collating and comparing the results, and (iii) reporting bias may have favoured the publication of studies identifying an association between extreme weather events and infectious diseases over studies finding no association, leading to a potential overestimation of the impact.

## Conclusion

Heavy rainfall and flooding events have been linked to the occurrence of infectious diseases across Europe. However, research in this area remains limited, highlighting a need for further studies as these extreme weather events become increasingly frequent. Strengthening the evidence base is critical to inform effective preparedness and response strategies. This review underscores the need for informed, adaptive public health strategies and calls for more research.

## Data Availability

All data are included in this published article and the supplementary materials.

## Supplementary Data

288000 Supplementary Material
