# Supplementary material for: Association between heavy rainfall and flooding events and infectious diseases in Europe: a rapid review, 2018 to 2025
Source: Euro Surveill. 2026 Aug 6;31(31):2500902. doi: 10.2807/1560-7917.ES.2026.31.31.2500902 (PMC13451863; doi:10.2807/1560-7917.ES.2026.31.31.2500902)
Supplement: Supplementary file 1 [file 25-00902_RISE_Supplement.pdf]

This supplementary material is hosted by *Eurosurveillance* as supporting information alongside the article 'Association between heavy rainfall and flooding events and infectious diseases in Europe: a rapid review, 2018 to 2025', on behalf of the authors, who remain responsible for the accuracy and appropriateness of the content. The same standards for ethics, copyright, attributions and permissions as for the article apply. Supplements are not edited by *Eurosurveillance* and the journal is not responsible for the maintenance of any links or email addresses provided therein.

Supplementary table S1: Detailed search strings

**- Embase -**	
#1	('flooding':ti,ab OR floods:ti,ab OR 'heavy rain*':ti,ab OR 'severe rain*':ti,ab) AND [humans]/lim AND [01-01-2018]/sd NOT [16-06-2025]/sd
#2	('infection'/de OR 'epidemic'/de OR infection* OR 'infectious disease*' OR 'communicable disease*' OR 'disease outbreak*' OR 'pathogenic disease*') AND [humans]/lim AND [01-01-2018]/sd NOT [16-06-2025]/sd
#3	#1 AND #2
#4	(((disease AND 'transmission'/de OR disease) AND transmission AND infectious OR 'infectious disease transmission' OR infection) AND transmission OR 'infection transmission') AND [humans]/lim AND [01-01-2018]/sd NOT [16-06-2025]/sd
#5	#1 AND #4
#6	(preparedness OR 'public health preparedness' OR response OR 'public health response') AND [humans]/lim AND [01-01-2018]/sd NOT [16-06-2025]/sd
#7	#3 AND # 6
#8	#3 OR #5 OR #7
**- PubMed -**	
#1	Flooding[tiab] OR floods[tiab] OR "heavy rain*"[tiab] OR "severe rain*"[tiab] AND ((humans[Filter]) AND (2018/1/1:2025/06/15[pdat]))"
#2	"Infections"[Mesh] OR "Disease Outbreaks"[Mesh] OR infection* OR "infectious disease*" OR "communicable disease*" OR "disease outbreak*" OR "pathogenic disease*" AND ((humans[Filter]) AND (2018/1/1:2025/06/15[pdat]))"
#3	#1 AND #2
#4	"Disease transmission, infectious"[MeSH] OR disease AND transmission AND infectious OR "infectious disease transmission" OR infection AND transmission OR "infection transmission" AND ((humans[Filter]) AND (2018/1/1:2025/06/15[pdat]))"
#5	#1 AND #4
#6	preparedness OR "public health preparedness" OR response OR "public health response" AND ((humans[Filter]) AND (2018/1/1:2025/06/15[pdat]))"
#7	#3 AND #6
#8	#3 OR #5 OR #7
**- Cochrane Library -**	
#1	Flooding OR floods OR heavy NEXT rain* OR severe NEXT rain* with Cochrane Library publication date Between Jan 2020 and Jun 2025
#2	MeSH descriptor: [Infections] explode all trees
#3	infection* OR infectious NEXT disease* OR communicable NEXT disease* OR disease NEXT outbreak* OR pathogenic NEXT disease* with Cochrane Library publication date Between Jan 2020 and Jun 2025
#4	#2 OR #3

#5	#1 AND #4 with Cochrane Library publication date Between Jan 2020 and Jun 2025
#6	MeSH descriptor: [Disease Transmission, Infectious] explode all trees
#7	disease AND transmission AND infectious OR infectious NEXT disease NEXT transmission OR infection AND transmission OR infection NEXT transmission with Cochrane Library publication date Between Jan 2020 and Jun 2025
#8	#6 OR #7 with Cochrane Library publication date Between Jan 2020 and Jun 2025
#9	#1 AND 8 with Cochrane Library publication date Between Jan 2020 and Jun 2025
#10	preparedness OR "public health preparedness" OR adaptation OR "public health adaptation" OR response OR "public health response" with Cochrane Library publication date Between Jan 2020 and Jun 2025
#11	#5 AND #10 with Cochrane Library publication date Between Jan 2020 and Jun 2025
#12	#5 OR #9 OR #11 with Cochrane Library publication date Between Jan 2020 and Jun 2025

Supplementary table S2: Modified critical appraisal tool

Adaptation of the Joanna Briggs Institute Critical Appraisal tool for Cross Sectional Analyses.

Criteria	Addressed bias	Applied scale	Scale	Comments
Were the criteria for inclusion in the sample clearly defined?	Q1	1 - 4	1 [Yes]<br>2 [No]<br>3 [Unclear]<br>4 [N/A]	
Were the study subjects and the setting described in detail?	Q2	1 - 4	1 [Yes]<br>2 [No]<br>3 [Unclear]<br>4 [N/A]	
Was the exposure measured in a valid and reliable way?	Q3	1 - 4	1 [Yes]<br>2 [No]<br>3 [Unclear]<br>4 [N/A]	'Measured' can be used interchangeably with 'described', or 'defined'.
Were objective, standard criteria used for measurement of the condition?	Q4	1 - 4	1 [Yes]<br>2 [No]<br>3 [Unclear]<br>4 [N/A]	
Were confounding factors identified?	Q5	1 - 4	1 [Yes]<br>2 [No]<br>3 [Unclear]<br>4 [N/A]	Expanded to include identification of limitation.
Were strategies to deal with confounding factors stated?	Q6	1 - 1	4 [N/A]	N/A for the purpose of a rapid review.

Were the outcomes measured in a valid and reliable way?	Q7	1 - 4	1 [Yes]<br>2 [No]<br>3 [Unclear]<br>4 [N/A]	Measured can be used interchangeably with described, or defined.
Was appropriate statistical analysis used?	Q8	1 - 1	4 [N/A]	N/A for the purpose of a rapid review.
